# Myocardial infarction following fast-track total hip and knee arthroplasty—incidence, time course, and risk factors: a prospective cohort study of 24,862 procedures

**DOI:** 10.1080/17453674.2018.1517487

**Published:** 2018-10-17

**Authors:** Pelle B Petersen, Henrik Kehlet, Christoffer C Jørgensen, on behalf of the Lundbeck Foundation Center for Fast-track Hip and Knee Replacement Collaborative Group

**Affiliations:** 1 Section for Surgical Pathophysiology, Rigshospitalet, University of Copenhagen, Copenhagen, Denmark;; 2 Lundbeck Foundation Center for Fast-track Hip and Knee Arthroplasty

## Abstract

Background and purpose — Acute myocardial infarction (MI) is a leading cause of mortality following total hip and knee arthroplasty (THA/TKA). The reported 30-day incidence of MI varies from 0.3% to 0.9%. However, most data derive from administration and insurance databases or large RCTs with potential confounding factors. We studied the incidence of and potential modifiable risk factors for postoperative MI in a large, multicenter optimized “fast-track” THA/TKA setting.

Patients and methods — A prospective cohort study was conducted on consecutive unselected elective primary unilateral THA and TKA, using prospective information on comorbidities and complete 90-day follow-up from the Danish National Patient Registry. Evaluation of discharge summaries and medical records was undertaken in cases of suspected MI. Logistic regression analyses were carried out for identification of preoperative risk factors.

Results — Of 24,862 procedures with a median length of stay 2 (IQR 2–3) days, 30- and 90-day incidence of MI was 31 (0.12%) and 48 (0.19%). Preoperative risk factors for MI ≤30 days were age >85 years (OR 7.4, 95% CI 2.3–24), insulin-dependent diabetes mellitus (IDDM) (3.6, CI 1.1–12), cardiovascular disease (2.4, CI 1.1–5.0) and hypercholesterolemia (2.3, CI 1.1–5.1). Of 31 patients with MI ≤30 days 9 were treated with vasopressors for intraoperative hypotension and 27 had postoperative anemia.

Interpretation — Fast-track THA and TKA had a low 30-day MI incidence. Focus on patients with age >85, IDDM, cardiovascular disease, and hypercholesterolemia may further reduce the 30-day incidence of MI. The role of postoperative anemia and intraoperative hypotension are other areas for further improvement

Acute myocardial infarction (MI) is a serious complication after total hip and knee arthroplasty (THA/TKA). Despite improvements in perioperative optimization and enhanced recovery throughout the last decades (Kehlet 2013), MI remains one of the most frequent causes of mortality following THA and TKA (Malviya et al. 2011, Kirksey et al. 2012, Bemenderfer et al. 2017, Jørgensen et al. 2017). Recent studies investigating the occurrence of postoperative MI found varying incidences both in hospital (0.2%–0.7%) (Menendez et al. 2015, Bemenderfer et al. 2017, Urban et al. 2017), after 30 days (0.3%–0.9%) (Singh et al. 2011, Belmont Jr et al. 2014, Pedersen et al. 2014, Thornqvist et al. 2014), and after 6 weeks (0.5% for THA and 0.2% for TKA) (Lalmohamed et al. 2012). In contrast, a recent large observational study on fast-track THA and TKA has shown a low 0.2% incidence of MI (Jørgensen et al. 2016b) and another observational study found a marked decrease in 30-day incidence of MI from around 0.9% before to 0.4% after implementation of a fast-track protocol (Khan et al. 2014). Furthermore, a recent study on early thromboembolic events after fast-track THA and TKA reported an incidence of only 0.07% for MI either in hospital or within the first postoperative week, with a minor increase to 0.10% within 30 days of surgery (Jørgensen et al. 2016a). However, most of the established evidence on incidence and risk factors for MI following THA and TKA derive from administrative databases relying on diagnostic codes or insurance data (Kirksey et al. 2012, Lalmohamed et al. 2012, Belmont Jr et al. 2014, Thornqvist et al. 2014, Menendez et al. 2015, Bemenderfer et al. 2017, Urban et al. 2017). These databases lack detailed information on perioperative care, which limits the ability to identify potential pathophysiological events leading to MI. Additionally, a novel comparison of NSQIP data and administrative databases has shown a low specificity of around 81% for both, and with chart review of a single complication as the “gold standard” (Etzioni et al. 2018). Consequently, we present results from a large prospective multicenter database on consecutive unselected elective primary THA and TKA in established fast-track settings with complete 90-day follow-up and chart review (Jørgensen and Kehlet 2013). We investigated the incidence of postoperative MI, potential modifiable preoperative risk factors, perioperative events disposing to the occurrence of MI, and MI related mortality after fast-track THA and TKA.

## Patients and methods

*This was an observational cohort study on prospectively collected consecutive unselected primary THA and TKA patients between January 2010 and October 2015. All patients were treated at 8 Danish orthopedic centers (> 200 procedures per year) with established fast-track protocols and reporting to the Lundbeck Foundation Centre for Fast-track Hip and Knee Replacement database (LCDB) (Jørgensen and Kehlet*^2013^*). The fast-track setup included regional anesthesia, multimodal opioid-sparing analgesia, in-hospital thromboprophylaxis, and early mobilization with discharge to own home (> 95%) when fulfilling a set of functional discharge criteria (Jørgensen et al.*^2013^*).*

Data on preoperative comorbidity and patient demographics were prospectively collected by patient reported questionnaires. Length of stay (LOS) (postoperative nights in hospital, including transfer to other departments and hospitals), 90-day readmission (≥ 1 night in hospital, and potentially surgically related) and mortality (Jørgensen and Kehlet 2013) were obtained from the Danish National Patient Registry (DNPR) (Schmidt et al. 2015). In case of LOS ≥5 days, readmission, or death within 90 days discharge summaries were analyzed. Furthermore, in all cases of suspected MI in the discharge summary or a diagnostic code (ICD10) related to MI in the DNPR, the complete medical records were reviewed. MI was classified according to the proposed EPCO definitions (Jammer et al. 2015). Additionally, in cases with MI ≤30 days results from blood samples and intraoperative anesthesia notes were acquired in order to identify any potential association between intraoperative events, anemia, and postoperative MI. Pre- and postoperative anemia were defined as hemoglobin (HB) < 13 g/dL for both male and females (Muñoz et al. 2017) and intraoperative hypotension was defined as an intraoperative systolic blood pressure <90 mmHg classified as dichotomous outcome (Sessler et al. 2017). Furthermore, in case of death the complete medical records on primary admission and eventual readmissions were obtained to establish cause of death and potential relation to index surgery. Finally, we obtained and analyzed discharge papers of patients with new postoperative administrations of anticoagulants through the Danish National Database of Reimbursed Prescriptions (Johannesdottir et al. 2012), to establish indication for the prescription. All discharge papers and medical records were initially analyzed by either the first (PBP) (patients from October 2013–November 2015) or the senior author (CCJ) (January 2010–September 2013). In cases of doubt the papers were evaluated by all authors until agreement was reached.

### Outcomes

Primary outcome was 30-day incidence of MI. Secondarily we investigated 90-day incidence, time course, and risk factors for MI.

### Statistics

No pre-study power analysis was conducted as we included all available procedures from the LCDB. Continuous data are presented as means (SD) or medians (IQR) and analyzed using Student’s t-test and non-parametric testing as appropriate. Categorical data are presented as actual number ((%) if n > 100, 95% confidence interval (CI)), and tested using χ2 or Fisher’s-exact test. We calculated risk factors with CI by multivariable logistic regression analyses in 4 models for MI ≤30 days and 4 similar models for MI ≤90 days. Variable selection was done based on clinically acknowledged risk factors (Mantilla et al. 2011, Belmont Jr et al. 2014) and directed acyclic graphs to reduce possible bias (Shrier and Platt 2008).

Due to the risk of mediation between cardiac disease, anticoagulant treatment, and hypercholesterolemia and between anticoagulant treatment, anemia, and cardiac disease, we constructed 4 models. The 1st included age (grouped in 5-year intervals), sex, diabetes, cardiovascular disease, and anemia. The 2nd included anticoagulant treatment but excluded cardiovascular disease and anemia. The 3rd included age, sex, diabetes, and hypercholesterolemia and the 4th included age, sex, diabetes, hypercholesterolemia, and cardiovascular disease. All variables were included as direct effects. Furthermore, to check for potential bias by including patients with more than 1 procedure (Bryant et al. 2006, Ravi et al. 2013) we performed a sensitivity analysis by analyzing incidence and risk factors from multiple logistic regression model 4 among the independent observations (patients with only 1 procedure). Statistical significance was defined as p < 0.05. All analyses were carried out using SPSS v.22 (IBM Corp, Armonk, NY, USA) and http://vassarstats.net/prop1.html for calculation of CI for proportions.

### Ethics, registration, funding, and potential conflicts of interest

No ethical approval was necessary due to the non-interventional study design. Permission to store and review patient data without prior informed consent was obtained from the Danish Data protection Agency (RH-2017-132) and the Danish National Board of Health (3-3013-56/2/EMJO). Furthermore, LCDB is registered on ClinicalTrials.gov (ID: NCT01515670) as an ongoing study registry for this type of studies.

The study was supported by a PhD grant to PBP from Lundbeckfonden (R230-2017-166), which took no part in organizing, analyzing, or writing the manuscript. Outside the submitted work HK is a member of the advisory board at “Rapid Recovery” by Zimmer Biomet and CCJ received speakers’ fees from “Rapid Recovery” by Zimmer Biomet.

## Results

**Figure 1.**
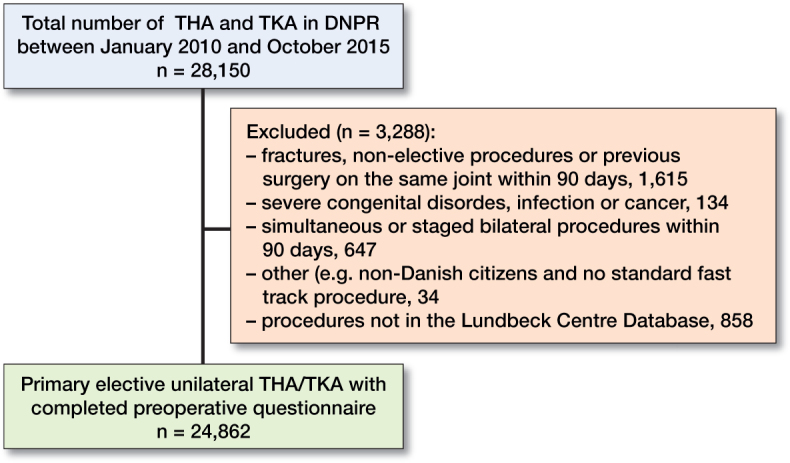
Flow of patients. DNPR = Danish National Patient Registry.

We included 24,862 procedures in 22,291 patients (97% of performed procedures) (Figure 1). Mean age was 68 (11) years and 58% were females (Table 1). Median LOS was 2 (2–3) days and 7.3% (CI 7.0–7.6) had a LOS ≥5 days. 30- and 90-day readmission rates were 5.6% (CI 5.3–5.8) and 7.6% (CI 7.3–7.9), respectively. Overall mortality was 0.15% (CI 0.11–0.21) within 30 days and 0.31% (0.25–0.39) within 90 days.

**Table 1.** Preoperative patient characteristics. Values are number (%) unless otherwise stated

	TKA		THA	
Characteristic	11,873	(47.8)	12,989	(52.2)
Age**^a^** (years)	68	(10)	68	(11)
< 50	559	(4.7)	857	(6.6)
50–60	2,135	(18)	1,926	(15)
61–65	1,790	(15)	1,808	(14)
66–70	2,374	(20)	2,669	(21)
71–75	2,279	(19)	2,378	(18)
76–80	1,641	(14)	1,874	(14)
81–85	818	(6.9)	1,025	(7.9)
> 85	277	(2.3)	452	(3.5)
Female sex	7,236	(61)	7,221	(56)
BMI				
< 18.5	44	(0.4)	132	(1.0)
18.5–24.9	2,305	(19)	4,411	(34)
25.0–29.9	4,528	(38)	5,309	(41)
30.0–34.9	3,118	(26)	2,335	(18)
35.0–39.9	1,298	(11)	549	(4.2)
≥ 40	524	(4.4)	172	(1.3)
Missing	56	(0.5)	81	(0.6)
Smoking	1,542	(13)	1,955	(15)
Missing	86	(0.7)	87	(0.7)
Alcohol >2 units daily	807	(6.8)	1,028	(7.9)
Missing	101	(0.9)	108	(0.8)
Social situation				
Living with others	7,918	(67)	8,597	(66)
Living alone	3,858	(33)	4,246	(33)
Nursing home. etc.	53	(0.4)	104	(0.8)
Missing	44	(0.4)	42	(0.3)
Preoperative anemia^b^	2,907	(25)	3,159	(24)
Missing	121	(1.0)	234	(1.8)
Use of walking aid	2,482	(21)	3,267	(25)
Missing	232	(2.0)	0	(0.0)
Hypertension	5,809	(49)	5,699	(44)
Missing	62	(0.5)	55	(0.4)
Hypercholesterolemia	4,779	(40)	4,656	(36)
Missing	70	(0.6)	69	(0.5)
Pharmacologically treated				
cardiac disease	1,691	(14)	1,740	(13)
Missing	130	(1.1)	81	(0.6)
pulmonary disease	1,042	(8.8)	1,109	(8.5)
Missing	78	(0.7)	58	(0.4)
psychiatric disorder	1,053	(8.9)	1,002	(7.7)
Missing	91	(0.8)	61	(0.5)
Anticoagulant treatment **^c^**	745	(6.3)	777	(6.0)
Missing	0	(0.0)	0	(0.0)
Prior cerebral stroke	744	(6.3)	752	(5.8)
Missing	179	(1.5)	170	(1.3)
Diabetes				
Insulin-dependent	300	(2.5)	202	(1.6)
Non-insulin-dependent	1,222	(10)	899	(6.9)
Missing	29	(0.2)	17	(0.1)

BMI = body mass index; THA = total hip arthroplasty;

TKA = total knee arthroplasty.

a mean (SD);

b Hb <13 g/dL

c Vitamin K antagonists or new oral anticoagulants.

The 30-day incidence of MI was 31 (0.12%, CI 0.08–0.17) and time to MI was median 4 (2–-8) days after surgery, with 13 (0.05%, CI 0.03–0.09) during primary admission. Of the MIs, 6 were diagnosed by rise in troponin combined with ECG abnormalities and 7 by troponin and symptoms of ischemia. In the remaining 18 patients, 5 MIs were verified by coronary angiography, 12 by percutaneous coronary intervention, and 1 by radiological evidence of new loss of viable myocardium.

**Figure 2.**
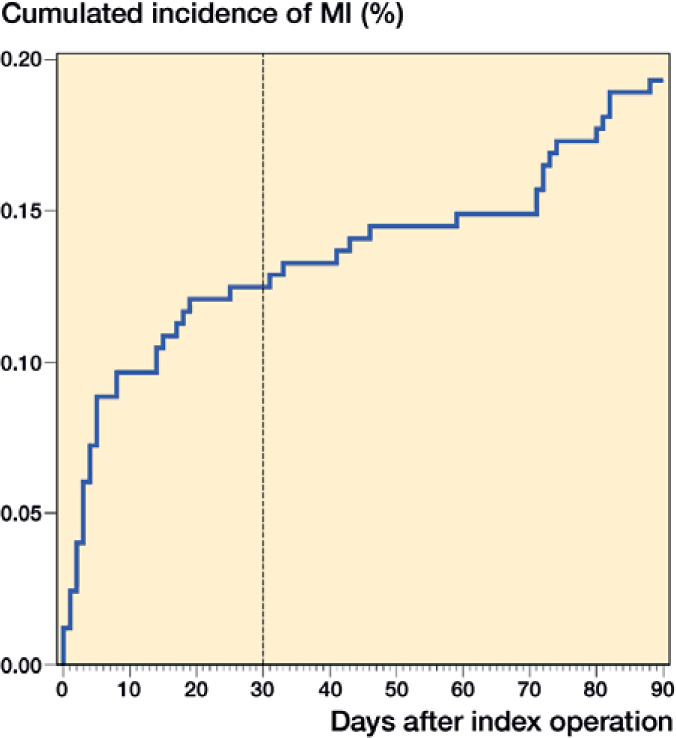
Cumulated incidence of MI in 24,862 THA and TKA procedures.

In addition, 17 (0.07%, CI 0.04–0.11) MIs occurred during days 30–90 resulting in a 90-day incidence of 48 (0.19%, CI 0.14–0.25) (Figure 2), similar between THA (n = 29, 0.22%, CI 0.15–0.32) and TKA (n = 19, 0.16%, CI 0.10–0.25) (p = 0.3). Median time to MI was 11 (3–56) days for MI ≤90 days. Prior to readmission with MI 1 patient had a readmission due to anemia 2 days prior to admission with MI (8 days postoperatively, LOS 1). No additional readmissions prior to MI occurred in the 47 remaining patients.

90-day mortality was 77 (0.31%, CI 0.25–0.39), of which proven or suspected MI was the primary cause of death in 5 (0.02%) patients. Of the 5 deaths caused by proven or suspected MI, 3 occurred during primary admission (postoperative days 2, 3, and 4) and 2 were after discharge (days 15 and 43). The 90-day mortality rate for postoperative MI patients was 6.3% (CI 2.2–17). 20 (0.08%, CI 0.05–0.12) patients died in their own home of unknown cause, with a median time to death of 64 (37–84) days.

When analyzing risk factors for MI ≤30 days, age of >85 years (OR 6.9–8.3) and insulin-dependent diabetes mellitus (IDDM) (OR 3.6–5.0) were statistically significantly associated with increased risk of MI regardless of the type of regression model. Preoperative cardiac disease (OR 2.4–2.8) and hypercholesterolemia (OR 2.3–2.7) were also significantly associated with MI in both models including these covariates (Table 2). In contrast, sex, use of anticoagulants, and preoperative anemia were not significantly associated with MI (Table 2). When assessing risk factors for MI ≤90 days the results were much alike although the association with hypercholesterolemia was statistically not significant when adjusting for cardiac disease (p = 0.06), while male sex was significantly associated with MI ≤90 days (OR 2.3–2.5) in all 4 models (Table 3).

**Table 2.** Multivariable logistic regressions for preoperative risk factors for MI within 30 days of surgery

	Model 1		Model 2		Model 3		Model 4	
Variable	OR (95% CI)	p-value	OR (95% CI)	p-value	OR (95% CI)	p-value	OR (95% CI)	p-value
Age		0.009		0.003		0.002		0.006
< 50	NA		NA		NA		NA	
50–60	NA		NA		NA		NA	
61–65	0.25 (0.03–2.1)	0.2	0.24 (0.03–2.0)	0.2	0.25 (0.03–2.1)	0.2	0.26 (0.03–2.2)	0.2
66–70	1	Ref	1	Ref	1	Ref	1	Ref
71–75	1.1 (0.35–3.3)	0.9	1.1 (0.36–3.4)	0.9	1.1 (0.36–3.4)	0.9	1.1 (0.35–3.3)	0.9
76–80	1.3 (0.43–4.2)	0.6	1.5 (0.47–4.5)	0.5	1.4 (0.47–4.5)	0.5	1.3 (0.43–4.2)	0.6
81–85	2.7 (0.85–8.4)	0.09	3.0 (0.94–8.3)	0.06	3.0 (0.95–9.3)	0.06	2.7 (0.85–8.5)	0.09
> 85	6.9 (2.2–21.9)	0.001	7.9 (2.5–24.7)	< 0.001	8.3 (2.6–26.3)	< 0.001	7.4 (2.3–23.5)	0.001
Male sex	1.6 (0.79–3.3)	0.2	1.8 (0.86–3.6)	0.1	1.8 (0.87–3.6)	0.1	1.6 (0.79–3.33)	0.2
Diabetes		0.04		0.03		0.07		0.07
Insulin-dependent	4.5 (1.3–15.3)	0.02	5.0 (1.5–16.7)	0.009	3.7 (1.1–12.6)	0.04	3.6 (1.1–12.4)	0.04
Non-insulin-dependent	0.69 (0.16–2.9)	0.6	1.3 (0.58–2.8)	0.6	0.59 (0.14–2.5)	0.5	0.56 (0.13–2.4)	0.4
Cardiovascular disease	2.8 (1.3–5.8)	0.006	–	–	–	–	2.4 (1.1–5.0)	0.02
Anemia**^a^**	1.2 (0.5–2.5)	0.7						
Hypercholesterolemia	–	–	–	–	2.7 (1.3–5.9)	0.01	2.3 (1.1–5.1)	0.03
Anticoagulant treatment**^b^**	–	–	1.4 (0.50–4.2)	0.5	–	–	–	–

Number of patients included in analysis differs from the total number of patients due to missing data. NA: not available due to lack of data.

a Hb <13 g/dL for both men and women.

b Vitamin K antagonists or new oral anticoagulants (NOAC).

**Table 3.** Multivariable logistic regressions for preoperative risk factors for MI within 90 days of surgery

	Model 1		Model 2		Model 3		Model 4	
Variable	OR (95% CI)	p-value	OR (95% CI)	p-value	OR (95% CI)	p-value	OR (95% CI)	p-value
Age		< 0.001		< 0.001		< 0.001		< 0.001
< 50	NA		NA		NA		NA	
50–60	0.13 (0.02–1.0)	0.05	0.12 (0.02–0.90)	0.04	0.13 (0.02–1.0)	0.05	0.14 (0.02–1.1)	0.06
61–65	0.14 (0.02–1.1)	0.06	0.13 (0.02–1.0)	0.05	0.14 (0.02–1.0)	0.06	0.14 (0.02–1.1)	0.06
66–70	1	Ref	1	Ref	1	Ref	1	Ref
71–75	0.78 (0.31–2.0)	0.6	0.81 (0.32–2.0)	0.6	0.81 (0.32–2.0)	0.6	0.78 (0.31–1.9)	0.6
76–80	1.2 (0.51–2.9)	0.7	1.3 (0.56–3.14)	0.5	1.3 (0.57–3.2)	0.5	1.2 (0.52–2.9)	0.6
81–85	2.2 (0.89–5.4)	0.09	2.5 (1.0–6.0)	0.05	2.5 (1.0–6.2)	0.04	2.3 (0.9–5.5)	0.07
> 85	5.0 (1.9–12.9)	0.001	5.9 (2.4–15.1)	< 0.001	6.3 (2.5–16.0)	< 0.001	5.5 (2.2–12.1)	< 0.001
Male sex	2.3 (1.3–4.3)	0.005	2.5 (1.4–4.5)	0.002	2.5 (1.4–4.5)	0.002	2.3 (1.3–4.1)	0.006
Diabetes		0.003		0.002		0.006		0.006
Insulin-dependent	4.4 (1.7–11.5)	0.002	5.0 (1.9–12.9)	0.001	3.9 (1.5–10.2)	0.006	3.9 (1.5–10.1)	0.006
Non-Insulin-dependent	0.42 (0.10–1.7)	0.2	0.47 (0.11–1.9)	0.3	0.38 (0.09–1.6)	0.2	0.36 (0.09–1.5)	0.2
Cardiovascular disease	3.0 (1.7–5.4)	< 0.001	–	–	–	–	2.7 (1.4–4.9)	0.001
Anemia**^a^**	1.2 (0.62–2.2)	0.6						
Hypercholesterolemia	–	–	–	–	2.2 (1.2–4.0)	0.01	1.8 (0.99–3.4)	0.06
Anticoagulant treatment**^b^**	–	–	1.7 (0.73–3.7)	0.2	–	–	–	–

For footnotes, see Table 2.

In-hospital postoperative hemoglobin (Hb) levels were available in 28 of the 31 patients with MI ≤30 days. Of these, 27 were anemic with a mean postoperative Hb of 9.9 (1.7) g/dL, and with a mean postoperative Hb reduction of 3.6 g/dL (CI 2.8–4.3). 10 MI patients with postoperative anemia also had preoperative anemia. Perioperative anesthesia notes were available in 24 cases with MI ≤30 days; 18 procedures were performed under spinal anesthesia. 11 patients developed intraoperative hypotension <90 mm/Hg, 6 during spinal anesthesia and 5 during general anesthesia. 9 of the intraoperative hypotensive patients required treatment with vasopressors, 5 in the spinal anesthesia group and 4 in the general anesthesia group. No other cardiac events were recorded during the surgical procedure.

Sensitivity analysis on the 19,793 patients with only 1 procedure showed similar results as mentioned above in the entire cohort. Thus, in the 19,793 patients LOS was median 2 (2–3) days, 7.6% (CI 7.2–7.9) had LOS ≥5 days and 7.9% (CI 7.5–8.3) had a readmission within 90 days. 26 patients (0.13%, CI 0.9–0.19) developed MI within 30 days and 41 (0.21%, 0.16–0.29) within 90 days. Risk factors for MI ≤30 days were age >85 years OR 6.8 (CI 1.9–24, p = 0.003), IDDM OR 4.5 (CI 1.3–16, p = 0.02), hypercholesterolemia OR 2.4 (CI 1.0–5.7, p = 0.05) and cardiovascular disease OR 2.3 (CI 1.0–5.2, p = 0.05). When analyzing the 5,069 patients with more than 1 procedure there was no difference. Median LOS was 2 (CI 2–3) days, 6.1% (CI 5.5–6.8%) had LOS ≥5 days, 90-day readmission rate was 6.6% (CI 6.0–7.4%) and 30-day incidence of MI was 0.10% (CI 0.04–0.23%) and was similar compared with the entire cohort. When comparing the 2 subgroups, 30-day MI incidence was similar (p = 0.7). However, patients with multiple procedures had a lower proportion of LOS ≥5 days (p < 0.001) and readmissions within 90 days (p = 0.003).

## Discussion

In this prospective cohort study on 24,862 consecutive unselected primary elective fast-track THA and TKAs we found a 30-day MI incidence of 0.12% with increased risk for age >85, IDDM, cardiovascular disease, and hypercholesterolemia in multivariable analysis. Furthermore, perioperative anemia and intraoperative hypotension was frequent among patients with postoperative MI.

Our MI incidence is lower than reported in studies on administrative databases reporting 30-day incidences of 0.25%–0.9% (Singh et al. 2011, Lalmohamed et al. 2012, Belmont Jr et al. 2014, Pedersen et al. 2014, Thornqvist et al. 2014). A recent comparison of administrative data and the clinical NSQIP registry have shown a low specificity of ≈ 81% for both sources of data compared with chart review (Etzioni et al. 2018), potentially underestimating the true incidence. Nonetheless, we found an in-hospital incidence rate of only 0.05%, considerably lower than previous data from such administrative and clinical registries with comparable cohorts but variable follow-up (Pulido et al. 2008, Malviya et al. 2011, Pedersen et al. 2014, Bemenderfer et al. 2017, Urban et al. 2017). Although, the numbers we found are comparable to previous fast-track THA and TKA studies (Malviya et al. 2011, Khan et al. 2014), previous studies lack detailed information on potential risk factors and events preceding MI.

Median time to MI ≤30 days was 4 (2–8) days and 11 (3–56) days for MI ≤90 days with increased cumulated incidence within the first 3 weeks. This is in concordance with previous data from the few THA- and TKA-specific studies, which report increased risk of MI within the first 2 weeks and up to 6 weeks for THA (Lalmohamed et al. 2012, Belmont Jr et al. 2014).

When evaluating preoperative risk factors for MI ≤90 days, age >85 years was found to be the most important risk factor for MI with OR of about 6. That increasing age is a risk factor for MI has been demonstrated in several studies (Belmont Jr et al. 2014, Menendez et al. 2015, Urban et al. 2017); however, we were unable to demonstrate any significant association between age and MI until age >85 years. That IDDM, cardiovascular disease, and hypercholesterolemia were found to be associated with MI in our study is unsurprising as these are well-known risk factors (Mantilla et al. 2011, Belmont Jr et al. 2014, Menendez et al. 2015). However, that male sex was clearly associated with 90- but not 30-day MI has to our knowledge not been shown previously. A previous investigation on early thromboembolic events including some of the same patients as our study suggested postoperative anemia as a possible pathogenic mechanism (Jørgensen et al. 2016a). Preoperative anemia is well known to dispose to postoperative anemia and blood transfusions (Goodnough et al. 2011), including in fast-track THA and TKA (Jans et al. 2018). In our study, preoperative anemia was not associated with MI. However, 12 of the 48 patients with MI had preoperative and 27 had postoperative anemia. Although conflicting results on anemia as a risk factor for postoperative MI have been discussed previously (Mantilla et al. 2011, Menendez et al. 2015), anemia can and should be prevented both pre- and postoperatively (Goodnough et al. 2013, Belmont Jr et al. 2014, Pujol-Nicolas et al. 2017), and increased focus on blood management remains a potential “low hanging fruit” for further improvement.

We also identified 11 cases of intraoperative hypotension of which 9 cases were treated with vasopressors. This occurred in 5 of 6 MI patients having general anesthesia in contrast to 6 of 18 patients having spinal anesthesia. Several studies from unspecific non-cardiac surgery have shown an association between intraoperative hypotension and risk of MI and mortality (Walsh et al. 2013, Roshanov et al. 2017, Sessler et al. 2017). Thus, an increased focus on avoiding a supply–demand mismatch during surgery may possibly further decrease the risk of MI. This hypothesis is also backed by a positive effect of an individualized blood pressure management strategy lowering postoperative organ dysfunction (Futier et al. 2017), although not specifically addressing MI. In addition, a recent study on etiology of MI after non-cardiac surgery found about 75% of MIs to be “demand” ischemia (type 2 myocardial infarctions) (Garcia et al. 2014). However, we do not know the incidence of intraoperative hypotension in patients who did not develop MI and, thus, further research on intraoperative hypotension and postoperative MI in THA and TKA is needed.

Our study has some methodological limitations. We only obtained health records from patients with LOS ≥5 days, readmission, and/or death within 90 days. Thus, a case of MI during primary admission with total LOS <5 days and without a diagnostic code of MI would be missed. However, that a patient with MI would be discharged within 4 days of surgery without a diagnostic code and no postoperative prescriptions of anticoagulants is unlikely. Although the LCDB aims at including all patients, 3% of the eligible population from the DNPR was missing in the LCDB (see Figure 1). However, we have previously shown similar postoperative outcomes in these patients (Jørgensen et al. 2013). Data on readmissions were obtained from the DNPR, which has shown varying reliability with regards to specific diagnostic codes (Vest-Hansen et al. 2013, Egholm et al. 2016) but >99% overall reliability with regards to somatic admission (Schmidt et al. 2015). Consequently, we obtained discharge summaries on all patients with LOS ≥5 and medical records including results from blood samples and intraoperative anesthesia records in case of MI to ensure best available information on incidence and pathophysiological mechanisms (Etzioni et al. 2018).

The declining autopsy rate in Denmark limits the availability of specific cause of death as seen in our study by 20 deaths of unknown causes. Consequently, although we obtained all healthcare records of patients dying ≤90 days of surgery to establish potential relation to surgery, we may underestimate the true MI incidence. Nonetheless, most deaths of unknown reason occurred >30 days postoperatively, and even if all 20 deaths were due MI, the 90-day MI rate would remain <0.3%. The lack of information on postoperative anemia and intraoperative hypotension in patients not developing MI precluded relevant statistical analyses. Likewise, Hb values at readmission were not consistently available, thus precluding conclusions on possible effects of post-discharge anemia and MI. Furthermore, our study design also impedes comparison on risk of MI with a non-surgical group as no matched control group was made.

Finally, about 10% of the patients had a second procedure during the study period, which may limit external validity of our results (Bryant et al. 2006). However, the incidence of and risk factors for MI among the patients with only 1 procedure or those with multiple primary arthroplasties was not different from the entire cohort. Additionally, leaving out patients with multiple primary arthroplasties may also introduce additional bias to postoperative outcomes (Ravi et al. 2013). Nonetheless, the inclusion of consecutive “real-life” patients from 8 different centers throughout Denmark without any selection bias increases the generalizability within fast-track THA and TKA.

In summary, the incidence of MI ≤30 days after fast-track THA and TKA was only 0.12%, increasing to 0.19% at 90 day. Age >85 years, IDDM, hypercholesterolemia, and cardiovascular disease were associated with MI ≤30 days. The roles of perioperative anemia and intraoperative hypotension as potential modifiable risk factors of developing MI remain areas for further research.  

PBP: Designing the work; acquisition, analysis and interpretation of data; drafting and revising the manuscript. HK: Designing the work, interpretation of data, revising the manuscript. CCJ: Designing the work, acquisition and interpretation of data, revising the manuscript.

## Notes

*Acta* thanks Keijo T Mäkelä and other anonymous reviewers for help with peer review of this study.

Belmont Jr P J, Goodman G P, Kusnezov N A, Magee C, Bader J O, Waterman B R, Schoenfeld A J. Postoperative myocardial infarction and cardiac arrest following primary total knee and hip arthroplasty: rates, risk factors, and time of occurrence. J Bone Joint Surg 2014; 96: 2025-31.
